# *N*′′-(4-Meth­oxy­phen­yl)-*N*,*N*,*N*′-trimethyl-*N*′-phenyl­guanidine

**DOI:** 10.1107/S1600536814005819

**Published:** 2014-03-22

**Authors:** Ioannis Tiritiris, Wolfgang Frey, Willi Kantlehner

**Affiliations:** a Fakultät Chemie/Organische Chemie, Hochschule Aalen, Beethovenstrasse 1, D-73430 Aalen, Germany; b Institut für Organische Chemie, Universität Stuttgart, Pfaffenwaldring 55, 70569 Stuttgart, Germany

## Abstract

In the title compound, C_17_H_21_N_3_O, the C—N bond lengths in the guanidine unit are 1.2889 (19), 1.3682 (19) and 1.408 (2) Å, indicating double- and single-bond character. The N—C—N angles are 115.10 (13), 119.29 (15) and 125.61 (14)°, showing a deviation of the CN_3_ plane from an ideal trigonal–planar geometry. In the crystal, non-classical C—H⋯O hydrogen bonds between methyl H atoms and meth­oxy O atoms are present, generating centrosymmetric dimers running in the [101] direction.

## Related literature   

For the crystal structures of *N*-methyl­ated di­phenyl­guanidines, see: Tanatani *et al.* (1998 ▶). For non-classical hydrogen bonds, see: Desiraju & Steiner (1999 ▶). For the crystal structure of *N*′′-(4-carbazol-9-ylphen­yl)-*N*,*N*′-diethyl-*N*,*N*′-di­phenyl­guani­dine, see: Tiritiris & Kantlehner (2013 ▶).
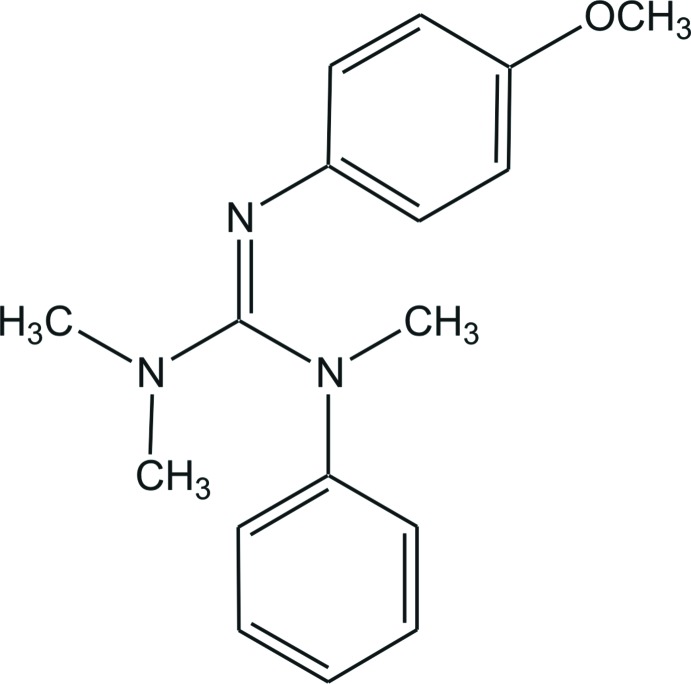


## Experimental   

### 

#### Crystal data  

C_17_H_21_N_3_O
*M* *_r_* = 283.37
Monoclinic, 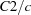

*a* = 26.691 (4) Å
*b* = 7.5135 (7) Å
*c* = 19.361 (2) Å
β = 125.412 (8)°
*V* = 3164.4 (7) Å^3^
*Z* = 8
Mo *K*α radiation
μ = 0.08 mm^−1^
*T* = 293 K
0.45 × 0.30 × 0.20 mm

#### Data collection  

Nicolet P3/F diffractometer
3817 measured reflections
3817 independent reflections
2770 reflections with *I* > 2σ(*I*)
3 standard reflections every 50 reflections intensity decay: 2%

#### Refinement  

*R*[*F* ^2^ > 2σ(*F* ^2^)] = 0.055
*wR*(*F* ^2^) = 0.161
*S* = 1.04
3817 reflections
195 parameters
H-atom parameters constrained
Δρ_max_ = 0.21 e Å^−3^
Δρ_min_ = −0.16 e Å^−3^

### 

Data collection: *XSCANS* (Siemens, 1996 ▶); cell refinement: *XSCANS*; data reduction: *SHELXTL* (Sheldrick, 2008 ▶); program(s) used to solve structure: *SHELXS97* (Sheldrick, 2008 ▶); program(s) used to refine structure: *SHELXL97* (Sheldrick, 2008 ▶); molecular graphics: *DIAMOND* (Brandenburg & Putz, 2005 ▶); software used to prepare material for publication: *SHELXL97*.

## Supplementary Material

Crystal structure: contains datablock(s) I, global. DOI: 10.1107/S1600536814005819/kp2467sup1.cif

Structure factors: contains datablock(s) I. DOI: 10.1107/S1600536814005819/kp2467Isup2.hkl

Supporting information file. DOI: 10.1107/S1600536814005819/kp2467Isup3.cml

CCDC reference: 991872

Additional supporting information:  crystallographic information; 3D view; checkCIF report

## Figures and Tables

**Table 1:** Hydrogen-bond geometry (Å, °)

*D*—H⋯*A*	*D*—H	H⋯*A*	*D*⋯*A*	*D*—H⋯*A*
C17—H17*A*⋯O1^i^	0.96	2.81	3.502 (2)	130

Symmetry code: (i) 
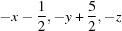
.

**Fig. 1.**
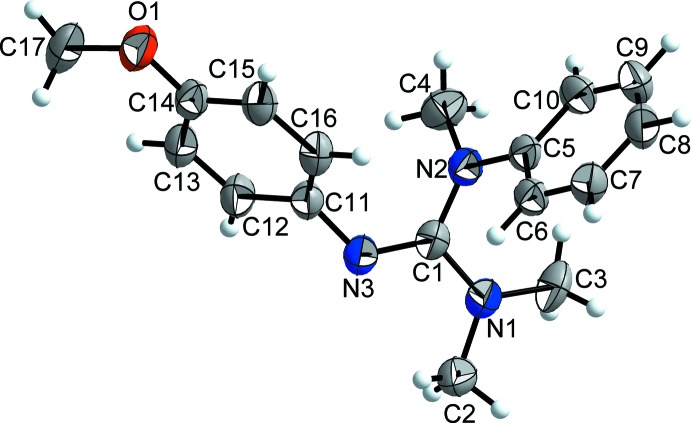
The structure of the title compound with atom labels and 50% probability displacement ellipsoids.

**Fig. 2.**
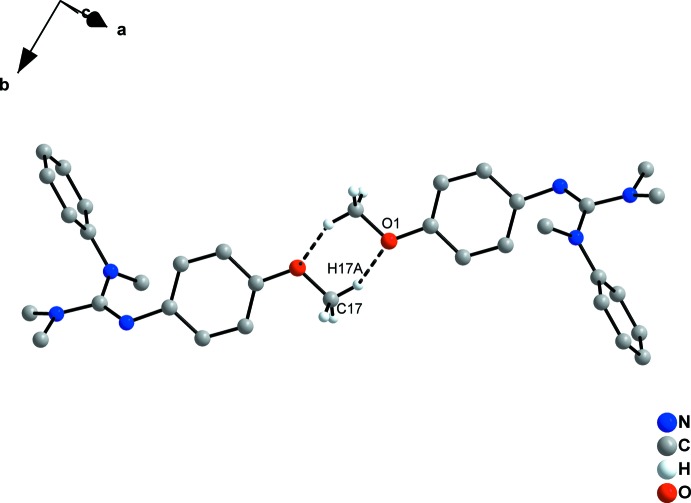
Non-classical C–H···O hydrogen bonds (indicated by dashed lines) between the methyl hydrogen atoms (H17A) and oxygen atoms (O1) of the methoxy groups, forming a centrosymmetric dimer.

**Fig. 3.**
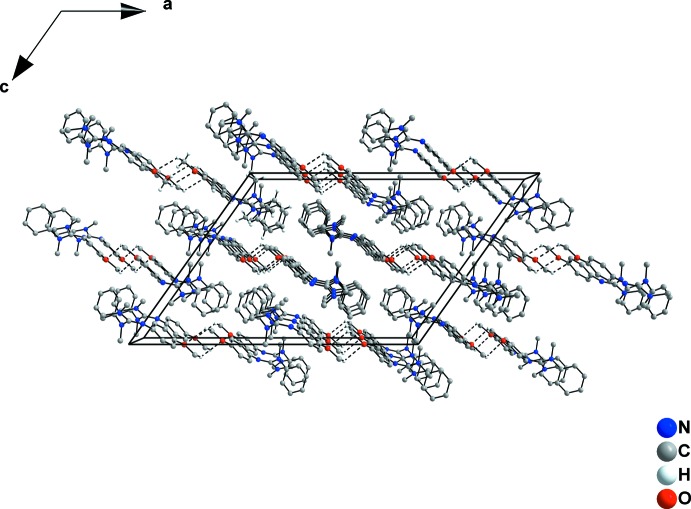
Packing diagram of the title compound in ac-plane. The hydrogen bonds are indicated by dashed lines.

## supplementary crystallographic information

### 1. Comment

The here presented title compound is similar to the structurally known compound *N''*-phenyl-*N*,*N*-dimethyl-*N'*,*N'*-methylphenyl- guanidine (Tanatani *et al.*, 1998). According to the structure analysis, the C1–N3 bond in the guanidine unit is 1.2889 (19) Å, indicating double bond character. The bond lengths C1–N2 = 1.408 (2) Å and C1–N1 = 1.3682 (19) Å are elongated and characteristic for C–N imine single bonds. The N–C1–N angles are 115.10 (13)° (N1–C1–N2), 125.61 (14)° (N2–C1–N3) and 119.29 (15)° (N1–C1–N3), showing a deviation of the CN_3_ plane from an ideal trigonal planar geometry (Fig. 1). Similar bond lengths and angles of the guanidine CN_3_ group have been found by structure analysis for *N''*-(4-carbazol-9-yl-phenyl)- *N*,*N'*-diethyl-*N*,*N'*-diphenyl-guanidine (Tiritiris & Kantlehner, 2013) and several *N*-methylated diphenylguanidines (Tanatani *et al.*, 1998). Non-classical C–H···O hydrogen bonds (Desiraju & Steiner, 1999) between methyl hydrogen atoms and oxygen atoms of the methoxy groups are present [*d*(H···O) = 2.81 Å] (Table 1), generating centrosymmetric dimers (Fig. 2 and Fig. 3) running in the direction [101].

### 2. Experimental

One equivalent of *N*,*N*-dimethyl-*N'*,*N'*-methylphenyl- chloroformamidinium-chloride (synthesized from *N*,*N*-dimethyl- *N'*,*N'*-methylphenylthiourea and phosgene) was reacted with one equivalent of 4-methoxyaniline (Sigma-Aldrich) in acetonitrile, in the presence of one equivalent of triethylamine, at 273 K. The obtained mixture consisting of the guanidinium chloride and triethylammonium chloride was reacted in the next step with an excess of an aqueous sodium hydroxide solution at 273 K. After extraction of the guanidine with diethyl ether from the water phase, the solvent was evaporated and the title compound was isolated in form of a colourless solid. Single crystals have been obtained by recrystallization from a saturated acetonitrile solution at 273 K.

### 3. Refinement

The hydrogen atoms of the methyl groups were allowed to rotate with a fixed angle around the C–N and C–O bond to best fit the experimental electron density, with *U*_iso_(H) set to 1.5 *U*_eq_(C) and d(C—H) = 0.96 Å. The H atoms in aromatic rings were placed in calculated positions with (C—H) = 0.93 Å. They were included in the refinement in the riding model approximation, with *U*_iso_(H) set to 1.2*U*_eq_(C).

### Crystal data

**Table:**

C_17_H_21_N_3_O	*F*(000) = 1216
*M**_r_* = 283.37	*D*_x_ = 1.190 Mg m^−^^3^
Monoclinic, *C*2/*c*	Mo *K*α radiation, λ = 0.71073 Å
Hall symbol: -C 2yc	Cell parameters from 32 reflections
*a* = 26.691 (4) Å	θ = 12.5–17.0°
*b* = 7.5135 (7) Å	µ = 0.08 mm^−^^1^
*c* = 19.361 (2) Å	*T* = 293 K
β = 125.412 (8)°	Block, colourless
*V* = 3164.4 (7) Å^3^	0.45 × 0.30 × 0.20 mm
*Z* = 8	

### Data collection

**Table:**

Nicolet P3/F diffractometer	*R*_int_ = 0.000
Radiation source: sealed tube	θ_max_ = 28.0°, θ_min_ = 1.9°
Graphite monochromator	*h* = −35→28
Wyckoff–Scan scans	*k* = 0→9
3817 measured reflections	*l* = 0→25
3817 independent reflections	3 standard reflections every 50 reflections
2770 reflections with *I* > 2σ(*I*)	intensity decay: 2%

### Refinement

**Table:**

Refinement on *F*^2^	Secondary atom site location: difference Fourier map
Least-squares matrix: full	Hydrogen site location: inferred from neighbouring sites
*R*[*F*^2^ > 2σ(*F*^2^)] = 0.055	H-atom parameters constrained
*wR*(*F*^2^) = 0.161	*w* = 1/[σ^2^(*F*_o_^2^) + (0.0907*P*)^2^ + 0.4749*P*] where *P* = (*F*_o_^2^ + 2*F*_c_^2^)/3
*S* = 1.04	(Δ/σ)_max_ < 0.001
3817 reflections	Δρ_max_ = 0.21 e Å^−^^3^
195 parameters	Δρ_min_ = −0.16 e Å^−^^3^
0 restraints	Extinction correction: *SHELXL97* (Sheldrick, 2008), Fc^*^=kFc[1+0.001xFc^2^λ^3^/sin(2θ)]^-1/4^
Primary atom site location: structure-invariant direct methods	Extinction coefficient: 0.0126 (12)

### Special details

**Table:**

Geometry. All e.s.d.'s (except the e.s.d. in the dihedral angle between two l.s. planes) are estimated using the full covariance matrix. The cell e.s.d.'s are taken into account individually in the estimation of e.s.d.'s in distances, angles and torsion angles; correlations between e.s.d.'s in cell parameters are only used when they are defined by crystal symmetry. An approximate (isotropic) treatment of cell e.s.d.'s is used for estimating e.s.d.'s involving l.s. planes.
Refinement. Refinement of *F*^2^ against ALL reflections. The weighted *R*-factor *wR* and goodness of fit *S* are based on *F*^2^, conventional *R*-factors *R* are based on *F*, with *F* set to zero for negative *F*^2^. The threshold expression of *F*^2^ > σ(*F*^2^) is used only for calculating *R*-factors(gt) *etc*. and is not relevant to the choice of reflections for refinement. *R*-factors based on *F*^2^ are statistically about twice as large as those based on *F*, and *R*-factors based on ALL data will be even larger.

### Fractional atomic coordinates and isotropic or equivalent isotropic displacement parameters (Å^2^)

**Table:**

	*x*	*y*	*z*	*U*_iso_*/*U*_eq_	
N1	0.08203 (6)	0.45184 (19)	0.19928 (10)	0.0707 (4)	
N2	0.05887 (6)	0.71681 (18)	0.12254 (7)	0.0580 (3)	
N3	−0.01532 (6)	0.56477 (18)	0.13348 (9)	0.0647 (4)	
C1	0.03887 (6)	0.5790 (2)	0.15042 (9)	0.0569 (4)	
C2	0.07023 (9)	0.3255 (3)	0.24493 (16)	0.0970 (7)	
H2A	0.0506	0.3854	0.2671	0.146*	
H2B	0.1084	0.2751	0.2908	0.146*	
H2C	0.0439	0.2325	0.2071	0.146*	
C3	0.12795 (8)	0.4022 (3)	0.18495 (13)	0.0820 (6)	
H3A	0.1185	0.2864	0.1593	0.123*	
H3B	0.1678	0.3999	0.2381	0.123*	
H3C	0.1279	0.4875	0.1480	0.123*	
C4	0.02692 (9)	0.7432 (3)	0.03201 (10)	0.0774 (5)	
H4A	0.0262	0.8677	0.0204	0.116*	
H4B	−0.0145	0.7000	0.0029	0.116*	
H4C	0.0478	0.6793	0.0128	0.116*	
C5	0.11327 (6)	0.8100 (2)	0.18048 (9)	0.0525 (3)	
C6	0.13389 (7)	0.8259 (2)	0.26485 (9)	0.0586 (4)	
H6	0.1117	0.7736	0.2828	0.070*	
C7	0.18709 (7)	0.9188 (2)	0.32194 (10)	0.0663 (4)	
H7	0.2004	0.9275	0.3781	0.080*	
C8	0.22101 (8)	0.9991 (3)	0.29729 (13)	0.0744 (5)	
H8	0.2570	1.0604	0.3363	0.089*	
C9	0.20037 (8)	0.9863 (3)	0.21424 (13)	0.0768 (5)	
H9	0.2225	1.0410	0.1967	0.092*	
C10	0.14755 (8)	0.8943 (2)	0.15616 (11)	0.0674 (4)	
H10	0.1345	0.8879	0.1000	0.081*	
C11	−0.05759 (6)	0.7075 (2)	0.09794 (9)	0.0570 (4)	
C12	−0.11896 (7)	0.6672 (2)	0.03552 (11)	0.0676 (4)	
H12	−0.1300	0.5498	0.0180	0.081*	
C13	−0.16389 (7)	0.7981 (3)	−0.00107 (11)	0.0683 (4)	
H13	−0.2044	0.7684	−0.0438	0.082*	
C14	−0.14870 (7)	0.9715 (2)	0.02570 (10)	0.0607 (4)	
C15	−0.08802 (7)	1.0149 (2)	0.08889 (10)	0.0626 (4)	
H15	−0.0774	1.1321	0.1072	0.075*	
C16	−0.04339 (7)	0.8840 (2)	0.12449 (10)	0.0610 (4)	
H16	−0.0030	0.9144	0.1671	0.073*	
O1	−0.19000 (6)	1.11075 (18)	−0.00565 (8)	0.0812 (4)	
C17	−0.25343 (8)	1.0690 (3)	−0.06419 (12)	0.0898 (7)	
H17A	−0.2772	1.1766	−0.0817	0.135*	
H17B	−0.2661	0.9915	−0.0376	0.135*	
H17C	−0.2597	1.0108	−0.1128	0.135*	

### Atomic displacement parameters (Å^2^)

**Table:**

	*U*^11^	*U*^22^	*U*^33^	*U*^12^	*U*^13^	*U*^23^
N1	0.0551 (7)	0.0659 (8)	0.0859 (10)	0.0101 (6)	0.0379 (7)	0.0117 (7)
N2	0.0533 (7)	0.0721 (8)	0.0512 (6)	0.0078 (6)	0.0318 (6)	0.0042 (6)
N3	0.0481 (6)	0.0664 (8)	0.0729 (8)	0.0038 (6)	0.0312 (6)	0.0037 (6)
C1	0.0486 (7)	0.0594 (8)	0.0585 (8)	0.0050 (6)	0.0286 (6)	0.0002 (6)
C2	0.0641 (10)	0.0750 (12)	0.1319 (18)	0.0057 (9)	0.0453 (12)	0.0345 (12)
C3	0.0589 (9)	0.0856 (12)	0.0863 (12)	0.0236 (9)	0.0333 (9)	−0.0041 (10)
C4	0.0842 (12)	0.0862 (12)	0.0534 (9)	0.0075 (10)	0.0350 (9)	0.0023 (8)
C5	0.0502 (7)	0.0610 (8)	0.0559 (7)	0.0110 (6)	0.0362 (6)	0.0060 (6)
C6	0.0544 (7)	0.0747 (9)	0.0563 (8)	0.0062 (7)	0.0376 (7)	0.0068 (7)
C7	0.0552 (8)	0.0834 (11)	0.0590 (8)	0.0057 (8)	0.0323 (7)	−0.0003 (8)
C8	0.0514 (8)	0.0812 (11)	0.0880 (12)	0.0003 (8)	0.0390 (8)	−0.0004 (9)
C9	0.0654 (10)	0.0875 (12)	0.1004 (13)	0.0015 (9)	0.0611 (10)	0.0110 (10)
C10	0.0699 (9)	0.0834 (11)	0.0705 (9)	0.0097 (8)	0.0531 (8)	0.0099 (8)
C11	0.0455 (7)	0.0688 (9)	0.0574 (8)	0.0028 (6)	0.0302 (6)	0.0023 (7)
C12	0.0486 (8)	0.0723 (10)	0.0731 (10)	−0.0024 (7)	0.0302 (7)	−0.0046 (8)
C13	0.0446 (7)	0.0873 (12)	0.0633 (9)	0.0040 (7)	0.0257 (7)	−0.0003 (8)
C14	0.0530 (8)	0.0815 (11)	0.0567 (8)	0.0136 (7)	0.0369 (7)	0.0079 (7)
C15	0.0586 (8)	0.0691 (9)	0.0671 (9)	0.0028 (7)	0.0405 (8)	−0.0083 (7)
C16	0.0467 (7)	0.0763 (10)	0.0577 (8)	0.0024 (7)	0.0290 (6)	−0.0083 (7)
O1	0.0646 (7)	0.0920 (9)	0.0876 (8)	0.0245 (6)	0.0445 (7)	0.0114 (7)
C17	0.0629 (10)	0.1322 (18)	0.0652 (10)	0.0366 (11)	0.0318 (9)	0.0137 (11)

### Geometric parameters (Å, º)

**Table:**

N1—C1	1.3682 (19)	C7—H7	0.9300
N1—C2	1.450 (3)	C8—C9	1.368 (3)
N1—C3	1.453 (2)	C8—H8	0.9300
N2—C5	1.4035 (19)	C9—C10	1.376 (3)
N2—C1	1.408 (2)	C9—H9	0.9300
N2—C4	1.4513 (19)	C10—H10	0.9300
N3—C1	1.2889 (19)	C11—C16	1.392 (2)
N3—C11	1.4133 (19)	C11—C12	1.393 (2)
C2—H2A	0.9600	C12—C13	1.386 (2)
C2—H2B	0.9600	C12—H12	0.9300
C2—H2C	0.9600	C13—C14	1.374 (3)
C3—H3A	0.9600	C13—H13	0.9300
C3—H3B	0.9600	C14—O1	1.3792 (19)
C3—H3C	0.9600	C14—C15	1.389 (2)
C4—H4A	0.9600	C15—C16	1.382 (2)
C4—H4B	0.9600	C15—H15	0.9300
C4—H4C	0.9600	C16—H16	0.9300
C5—C6	1.394 (2)	O1—C17	1.423 (2)
C5—C10	1.399 (2)	C17—H17A	0.9600
C6—C7	1.381 (2)	C17—H17B	0.9600
C6—H6	0.9300	C17—H17C	0.9600
C7—C8	1.383 (2)		
			
C1—N1—C2	119.01 (14)	C8—C7—H7	119.3
C1—N1—C3	120.83 (15)	C9—C8—C7	118.47 (17)
C2—N1—C3	116.97 (15)	C9—C8—H8	120.8
C5—N2—C1	120.47 (12)	C7—C8—H8	120.8
C5—N2—C4	120.59 (13)	C8—C9—C10	121.32 (15)
C1—N2—C4	118.38 (14)	C8—C9—H9	119.3
C1—N3—C11	121.50 (14)	C10—C9—H9	119.3
N3—C1—N1	119.29 (15)	C9—C10—C5	120.83 (15)
N3—C1—N2	125.61 (14)	C9—C10—H10	119.6
N1—C1—N2	115.10 (13)	C5—C10—H10	119.6
N1—C2—H2A	109.5	C16—C11—C12	117.32 (14)
N1—C2—H2B	109.5	C16—C11—N3	124.97 (13)
H2A—C2—H2B	109.5	C12—C11—N3	117.57 (15)
N1—C2—H2C	109.5	C13—C12—C11	121.54 (17)
H2A—C2—H2C	109.5	C13—C12—H12	119.2
H2B—C2—H2C	109.5	C11—C12—H12	119.2
N1—C3—H3A	109.5	C14—C13—C12	120.06 (15)
N1—C3—H3B	109.5	C14—C13—H13	120.0
H3A—C3—H3B	109.5	C12—C13—H13	120.0
N1—C3—H3C	109.5	C13—C14—O1	124.56 (14)
H3A—C3—H3C	109.5	C13—C14—C15	119.57 (15)
H3B—C3—H3C	109.5	O1—C14—C15	115.87 (16)
N2—C4—H4A	109.5	C16—C15—C14	120.01 (16)
N2—C4—H4B	109.5	C16—C15—H15	120.0
H4A—C4—H4B	109.5	C14—C15—H15	120.0
N2—C4—H4C	109.5	C15—C16—C11	121.46 (14)
H4A—C4—H4C	109.5	C15—C16—H16	119.3
H4B—C4—H4C	109.5	C11—C16—H16	119.3
C6—C5—C10	117.68 (15)	C14—O1—C17	117.52 (16)
C6—C5—N2	120.15 (13)	O1—C17—H17A	109.5
C10—C5—N2	122.15 (13)	O1—C17—H17B	109.5
C7—C6—C5	120.37 (14)	H17A—C17—H17B	109.5
C7—C6—H6	119.8	O1—C17—H17C	109.5
C5—C6—H6	119.8	H17A—C17—H17C	109.5
C6—C7—C8	121.31 (16)	H17B—C17—H17C	109.5
C6—C7—H7	119.3		
			
C11—N3—C1—N1	167.43 (14)	C7—C8—C9—C10	−0.8 (3)
C11—N3—C1—N2	−12.8 (2)	C8—C9—C10—C5	−0.2 (3)
C2—N1—C1—N3	−12.6 (2)	C6—C5—C10—C9	1.3 (2)
C3—N1—C1—N3	146.64 (17)	N2—C5—C10—C9	179.28 (15)
C2—N1—C1—N2	167.62 (17)	C1—N3—C11—C16	−44.6 (2)
C3—N1—C1—N2	−33.2 (2)	C1—N3—C11—C12	139.97 (16)
C5—N2—C1—N3	128.72 (16)	C16—C11—C12—C13	2.3 (3)
C4—N2—C1—N3	−59.8 (2)	N3—C11—C12—C13	178.05 (15)
C5—N2—C1—N1	−51.48 (19)	C11—C12—C13—C14	−1.8 (3)
C4—N2—C1—N1	120.04 (16)	C12—C13—C14—O1	−179.03 (15)
C1—N2—C5—C6	−27.5 (2)	C12—C13—C14—C15	0.7 (2)
C4—N2—C5—C6	161.16 (15)	C13—C14—C15—C16	−0.1 (2)
C1—N2—C5—C10	154.51 (14)	O1—C14—C15—C16	179.66 (14)
C4—N2—C5—C10	−16.8 (2)	C14—C15—C16—C11	0.6 (2)
C10—C5—C6—C7	−1.4 (2)	C12—C11—C16—C15	−1.6 (2)
N2—C5—C6—C7	−179.44 (14)	N3—C11—C16—C15	−177.09 (14)
C5—C6—C7—C8	0.4 (2)	C13—C14—O1—C17	5.9 (2)
C6—C7—C8—C9	0.6 (3)	C15—C14—O1—C17	−173.79 (14)

### Hydrogen-bond geometry (Å, º)

**Table:**

*D*—H···*A*	*D*—H	H···*A*	*D*···*A*	*D*—H···*A*
C17—H17*A*···O1^i^	0.96	2.81	3.502 (2)	130

Symmetry code: (i) −*x*−1/2, −*y*+5/2, −*z*.
